# Fixed-Time Global Sliding Mode Control for Parallel Robot Mobile Platform with Prescribed Performance

**DOI:** 10.3390/s25051584

**Published:** 2025-03-05

**Authors:** Aojie Wang, Guoqin Gao, Xue Li

**Affiliations:** School of Electrical and Information Engineering, Jiangsu University, Zhenjiang 212013, China; 2212207068@stmail.ujs.edu.cn (A.W.); 2112207002@stmail.ujs.edu.cn (X.L.)

**Keywords:** trajectory tracking, global sliding mode, fixed time control, prescribed performance control

## Abstract

A fixed-time global sliding mode control with prescribed performance is proposed for the varying center of mass parallel robot mobile platform with model uncertainties and external disturbances to improve the global robustness and convergence performance of the model, and reduce overshoots. Firstly, kinematic and dynamic models of the parallel robot mobile platform with a varying center of mass are established. A reference velocity controller for the mobile platform system’s outer loop is designed using the back-stepping method, which provides the expected reference velocity for the inner loop controller. Secondly, to improve the global robustness and convergence performance of the system, a fixed-time global sliding mode control algorithm in the inner loop of the system is designed to eliminate the reaching phase of sliding mode control and ensure that the system converges quickly within a fixed time. Meanwhile, by designing a performance function to constrain the system errors within the performance boundary further, the fixed-time global sliding mode control with prescribed performance is implemented to reduce overshoots of the system. Then, the Lyapunov stability of the proposed method is proved theoretically. Finally, the effectiveness and superiority of the proposed control method are verified by simulation experiments.

## 1. Introduction

Steel box girders are commonly used in the construction of long-span bridges. To extend their service life, sandblasting and derusting are needed. Existing wall-climbing robots struggle to effectively apply treatments to the complex curved surfaces of U-shaped ribs, and overhead crane sandblasting and derusting robots find it difficult to meet the flexible mobility requirements of bridge construction. To this end, our research group developed a sandblasting and derusting parallel robot (SDPR, Mute Technology Co., Ltd., Wuhan, China) [1] based on the Stewart parallel mechanism [2]. The parallel robot consists of a mobile platform, a parallel mechanism, and a lifting mechanism. During sandblasting and derusting operations, the movement of the lifting mechanism leads to variations in the mass of the mobile platform, causing changes in the inertia matrix in this dynamic model, which in turn affects its trajectory tracking control performance. Due to the changes in its inertia matrix and inevitable errors in the modeling process, the accuracy of system model of the mobile platform is uncertain. Furthermore, during the sandblasting and rust removal operation, the mobile platform is also susceptible to external interference, such as slippage.

Improving the robustness and convergence performance and reducing overshoots for varying mass mobile platforms with model uncertainties and external disturbances to achieve high-performance trajectory tracking control remains a significant challenge in the field of control [3,4,5].

Mobile platforms are a class of multi-input and multi-output nonlinear systems characterized by non-holonomic constraints, which arise from the inherent motion constraints of robot systems. A non-holonomic constraint restricts instantaneous motion in certain directions, posing challenges such as nonlinearity and constrained motion in trajectory tracking [6,7]. At present, several control methods have been proposed for trajectory tracking control in these systems, including backstepping control [8], model predictive control (MPC) [9], fuzzy control [10], synergetic control [11], and sliding mode control (SMC) [12]. Backstepping control has to know all system states and apply a recursive algorithm in the strict feedback form [13]. Model predictive control struggles to describe unstable systems and has difficulty recognizing online models. and the performance of MPC is highly dependent on the accuracy of the system model [14]. Fuzzy control faces challenges in complex multi-input systems, and the number of fuzzy rules increases exponentially with the number of system inputs, which could cause fuzzy control to be unimplementable [15]. Both synergetic control and sliding mode control use the same principle to drive the system to operate toward the manifold; both control methods benefit from order reduction [16]. Synergetic control operates at a constant switching frequency, and synergetic control requires a relatively lower bandwidth in the controller [17]. Compared with sliding mode control, synergetic control requires comparatively more complex calculations. Synergetic control requires a complete model of the controlled system and cannot suppress the system uncertainties and external disturbances [18]. Compared with synergetic control, sliding mode control is more robust and insensitive to system parameter changes. Therefore, this paper studies the tracking control of mobile platforms based on sliding mode control.

Variable structure controllers with sliding mode control were first proposed in the early 1950s. Essentially, the sliding mode control system drives the state of a system to reach a sliding surface in the phase plane and then keeps the state sliding in the subsequence. Due to its simple structure and good robustness to uncertainties, sliding mode control is widely used in uncertain systems, such as robot manipulators, aircraft, underwater vehicles, flexible space structures, electrical motors, power systems and automotive engines [19,20]. Chen [21] proposed a robust tracking control strategy predicated on a nonlinear disturbance observer, which was developed for a self-balancing mobile robot with external unknown disturbances. A nonlinear disturbance observer was devised to estimate the unknown disturbances in the self-balancing mobile robot. Concurrently, a sliding mode controller was designed to actualize precise trajectory tracking control for the robot, thereby enhancing its performance and stability in the face of uncertain environmental conditions. Traditional sliding mode control is robust to system uncertainty parameters and external disturbances only in the sliding phase, not in the reaching phase. To address the shortcomings of traditional sliding mode control, some scholars have proposed a global sliding mode control (GSMC) method [22,23]. The GSMC method ensures that the sliding mode surface is on the state trajectory from the beginning of the experiment by eliminating the reaching phase so that the system reaches global robustness. Chu et al. [24] proposed a global sliding mode controller based on a double hidden layer recurrent neural network (DHLRNN) for the trajectory tracking control problem of multi-input universal dynamic systems with external disturbances and system uncertainties. By designing a double hidden layer structure to better approximate the unknown function and a global sliding mode to make the system’s response globally robust. Zhang et al. [25] proposed an adaptive fuzzy-based global sliding mode control strategy for quadrotor unmanned aerial vehicles with parameter uncertainties and external disturbances. This approach eliminates the reaching phase of sliding mode control, counteracts system uncertainties online, and enhances the system’s robustness. In this paper, a global sliding mode controller is designed for a varying-mass mobile platform of parallel robots with model uncertainties and external disturbances.

The traditional sliding mode control methods typically achieve asymptotic stability by designing linear sliding surfaces, and the convergence time in these methods tends to be infinite. In the evolution of system dynamics, the closer to the equilibrium, the slower the state convergence [26]. To achieve faster convergence speeds, researchers have proposed a finite-time control theory. Liu et al. [27] studied the finite-time stability conditions for switched linear parameter-varying systems and designed a finite-time robust sliding mode controller. Mobayen [28] proposed a recursive terminal sliding mode control method for a class of nonholonomic systems with external disturbances to realize the finite-time stability of the system. Guo et al. [29] proposed a finite-time sliding mode control method, which not only realizes trajectory tracking and control of wheeled mobile robots with parameter uncertainties and disturbances but also improves the convergence speed of trajectory tracking errors. However, in practical engineering applications, accurate initial states may not be obtained or the initial states may be too large. The upper bound of the convergence time of finite-time control is related to the initial state of the system, which can impact the control performance of the system. To this end, Polyakov [30] first proposed the fixed-time stability theory, proving that the system convergence time in fixed-time control is independent of the initial state of the system according to numerical simulations. Fathollahi and Andresen [31] proposed an adaptive fixed-time control strategy to enhance the transient stability of power networks, addressing both single-machine systems connected to an infinite bus (SMIB) and multi-machine power systems operating under parametric uncertainties. Feng et al. [32] developed a fixed-time sliding mode control strategy incorporating disturbance observers to address robust stabilization in underactuated two-degree-of-freedom systems subject to multiple external disturbances. Saim et al. [33] developed a fault tolerant control based on an adaptive non-singular fixed-time terminal sliding mode controller for robot arm trajectory tracking problems with uncertainties, external disturbances, and actuator failures to achieve the rapid convergence of the system in a fixed time. Guo et al. [34] designed a fixed-time trajectory tracking controller based on a terminal sliding surface, which enabled a wheeled mobile robot with unknown camera parameters to track the upper reference trajectory in a fixed time. Control systems designed based on the theory of fixed time stability can achieve state trajectory convergence to the equilibrium point within a fixed time, and the settling time does not depend on the initial conditions of the system. This paper aims to incorporate the theory of fixed time control into the design of global sliding mode control.

While the aforementioned control methods can enhance the system’s convergence performance, they often overlook its transient performance, particularly the maximum overshoot of the system. To address this issue, Bechlioulis and Rovithakis [35] first proposed a method of prescribed performance, which introduced a performance function to constrain the convergence errors in the control system. For the problem of control in uncertain pure feedback nonlinear systems, Li and Liu [36] proposed a prescribed performance control scheme, causing the system’s state to vary within specified constraints. Liu et al. [37] designed a performance function that describes the convergence speed and maximum overshoot of the system for uncertain nonlinear systems with external disturbances and actuator failures and designed a tracking controller based on this to ensure the asymptotic convergence of the system. Sai et al. [38] proposed a continuous fixed-time terminal sliding mode control method for robot arm control problems with uncertainties and designed a fixed-time performance function to ensure that the uncertain robot trajectory tracking control converges in a fixed time and the tracking error is within the prescribed performance boundary. Bu et al. [39] designed a new performance function for the tracking control problem in air-breathing hypersonic vehicles to allow the system to have a transient performance with small overshoots. However, these methods generally do not consider the impact of uncertain disturbances on the system in the reaching phase. This paper introduces a novel predefined performance fixed-time global sliding mode controller for a varying mass mobile platform of parallel robotics with model uncertainties and external disturbances. By integrating the theory of predefined performance, this design aims to achieve high-performance trajectory tracking control.

Based on the above discussion, this paper introduces a fixed-time global sliding mode control method with a prescribed performance for trajectory tracking control within the mobile platform used by the sandblasting and derusting parallel robot (SDPR). The principal contributions of this paper are as follows:

1. A fixed-time global sliding mode strategy is proposed for use in the inner loop of the system. The strategy eliminates the reaching stage of sliding mode control by adding an auxiliary function to the sliding mode variable, which improves the global robustness of the system, and the global sliding mode control combined with the fixed time theory enables the mobile platform system to converge in a fixed time, improving the system’s convergence performance.
2. The prescribed performance control method is applied to constrain the error convergence characteristics of the inner loop controller and to reduce the overshoot of the tracking errors, which guarantees a good transient performance of the mobile platform system.

The structure of this paper is as follows. Section 2 describes the construction of a kinematic model and dynamic model of the varying mass of the mobile SDPR platform. In Section 3, a speed controller is designed based on the kinematic model of a vehicle-like robot, in the form of a mobile platform, to provide a reference input for the inner loop controller. Subsequently, a fixed-time global sliding mode null algorithm with a preset performance is introduced. Section 4 presents simulation experimental data and results, thereby substantiating the efficacy of the proposed method.

## 2. Problem Formulation and Preliminaries

### 2.1. Kinematic Model of the Mobile Platform of SDPR

**Figure 1:**
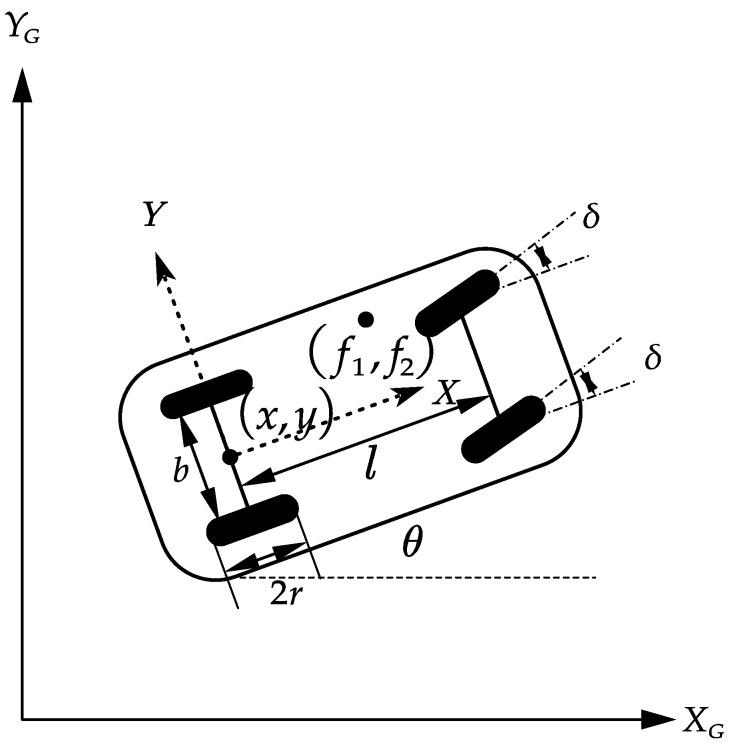
Vehicle-type mobile platform structure.

The research object of this article is a car-like mobile platform. A schematic structural diagram of the mobile platform with varying mass is shown in Figure 1, where the coordinates (x, y) represent the midpoint of the two rear wheels of the mobile platform in the global coordinate system; this midpoint serves as the reference point for trajectory tracking using the mobile platform. We designate this point as the origin coordinate to establish a local coordinate system on the mobile platform.

We utilized a vector $q={\left[x\;y\;\theta \;\delta \;\varphi \right]}^{T}$ to represent the position of the mobile platform in the global coordinate system, where *θ* is the angle between the direction of motion of the mobile platform and the direction of the *x*-axis, δ is the angle of the front wheel, *φ* is the angular velocity of the front wheel, *r* is the radius of the wheel, *b* is the distance between the two rear wheels, *l* is the distance between the front and rear wheel axles, and (*f_1_*, *f_2_*) is the position of the varying mass mobile platform in the local coordinate system. Assuming no lateral slip in the mobile platform, we can derive the following kinematic equations:

(1) $$\dot{q}=\left[\begin{array}{c} \dot{x}\\ \dot{y}\\ \dot{\theta }\\ \dot{\delta }\\ \dot{\varphi } \end{array}\right]=\left[\begin{array}{cc} \cos\theta & 0\\ \sin\theta & 0\\ \frac{\tan\varphi }{l} & 0\\ 0 & 1\\ \frac{1}{r} & 0 \end{array}\right]\left[\begin{array}{c} v\\ \omega \end{array}\right]=J(q)V$$

Assuming that the mobile platform is not disturbed by lateral slip, the non-holonomic constraints of the mobile platform are expressed as follows:

(2) $$\left\{\begin{array}{l} \dot{x}\sin\theta -\dot{y}\cos\theta =0\\ \dot{x}\sin(\theta +\varphi )-\dot{y}\cos(\theta +\varphi )-l\dot{\theta }\cos\varphi =0 \end{array}\right.$$

After simplification, the matrix can be rewritten as follows:

(3) $$H(q)\;\dot{q}=0$$

where

(4) $$H(q)=\left[\begin{array}{ccccc} \sin\theta & -\cos\theta & 0 & 0 & 0\\ \sin(\theta +\varphi ) & -\cos(\theta +\varphi ) & l\cos\varphi & 0 & 0 \end{array}\right]$$

### 2.2. Dynamic Model of the Mobile Platform of SDPR

The coordinates in the local coordinate system for the mobile platform’s mass are (*f*_1_ and *f*_2_), and the coordinates in the global coordinate system are ($x_{G}$ and $y_{G}$). Under the global coordinate system, the geometric position relationship equation between the mobile platform’s centroid position coordinates (*x*_G_ and *y*_G_) and the trajectory tracking reference point coordinates (*x* and *y*) are established:

(5) $$\left\{\begin{array}{l} x_{G}=x+f_{1}\sin\theta +f_{2}\cos\theta \\ y_{G}=y-f_{1}\cos\theta +f_{2}\sin\theta \end{array}\right.$$

According to the Lagrange method, the relationship between the wheel moment of the mobile platform and its position, speed, and acceleration can be analyzed, and a dynamic model of the mobile platform can be established. The Lagrange function (L) is defined as the difference between kinetic energy (T) and potential energy (P). The Lagrange equation can be expressed as follows:

(6) $$\frac{d}{dt}(\frac{\vartheta L}{\vartheta \dot{q}})-\frac{\vartheta L}{\vartheta q}=N(q)\;\tau +H{\left(q\right)}^{T}\gamma$$

where $H\left(q\right)\in R^{2X5}$ is the nonholonomic constraint matrix, $\gamma \in R^{2}$ is the Lagrange multiplier, and $\tau \in R^{2}$ is the moment output matrix. The total kinetic energy L of the mobile platform can be expressed as follows:

(7) $$L=L_{p}+L_{\omega }$$

where *L*_p_ represents the kinetic energy of the mobile platform body of the parallel robot mobile platforms with varying mass, and *L*_ω_ represents the kinetic energy of the driving and steering wheels of the mobile platform; they are expressed as follows:

(8) $$\begin{array}{l} L_{p}=\frac{1}{2}m_{p}({\dot{x}}_{G}^{2}+{\dot{y}}_{G}^{2})+\frac{1}{2}I_{p}{\dot{\theta }}^{2}\\ L_{\omega }=\frac{1}{2}m_{\omega }({\dot{x}}_{rf\omega }^{2}+{\dot{y}}_{rf\omega }^{2})+\frac{1}{2}m_{\omega }({\dot{x}}_{lf\omega }^{2}+{\dot{y}}_{lf\omega }^{2})+\frac{1}{2}m_{\omega }({\dot{x}}_{rr\omega }^{2}+{\dot{y}}_{rr\omega }^{2})\\ \;\;\;+\frac{1}{2}m_{\omega }({\dot{x}}_{lr\omega }^{2}+{\dot{y}}_{lr\omega }^{2})+I_{\omega }{\left(\dot{\theta }+\dot{\delta }\right)}^{2}+I_{\omega }{\dot{\theta }}^{2}+2I_{\omega }{\dot{\varphi }}^{2} \end{array}$$

The mobile platform does not consider the gravitational potential energy *P* = 0, so the Lagrange function *L* can be described as the sum of the kinetic energy of the car body *L_p_*, the kinetic energy of the front wheel *L_fw_*, and the kinetic energy of the rear wheel *L_rw_*. These terms can be substituted into Equation (6) to obtain the sandblasting and derusting parallel robot mobile platform standard dynamic model:

(9) $$M(q)\;\ddot{q}+C(q,\dot{q})\;\dot{q}=N(q)\;\tau +H^{T}(q)\;\gamma$$

where $M(q)\in R^{5\times 5}$ is the inertia matrix, $C(q,\dot{q})\in R^{5\times 5}$ is the Coriolis force term, and $N(q)\in R^{5\times 2}$ is the input conversion matrix. The elements in (9) are defined as follows:

$$\begin{array}{c} M(q,\dot{q})=\left[\begin{array}{ccccc} m & 0 & m13 & 0 & 0\\ 0 & m & m23 & 0 & 0\\ m13 & m23 & l\theta & 0 & 0\\ 0 & 0 & 2I\omega & 0 & 0\\ 0 & 0 & 0 & 0 & 0 \end{array}\right],C(q,\dot{q})=\left[\begin{array}{ccccc} 0 & 0 & v13 & 0 & 0\\ 0 & 0 & v23 & 0 & 0\\ 0 & 0 & 0 & 0 & 0\\ 0 & 0 & 0 & 0 & 0\\ 0 & 0 & 0 & 0 & 0 \end{array}\right]N(q)=\left[\begin{array}{cc} \cos\theta & 0\\ \sin\theta & 0\\ l\sin\varphi \cos\varphi & 0\\ 0 & 1\\ 1 & 0 \end{array}\right],\\ I=I_{p}+2m_{\omega }\left(2b^{2}+l^{2}\right)+4I_{\omega }+m_{p}\left({f_{1}}^{2}+{f_{2}}^{2}\right),m=m_{p}+4m_{\omega },\\ I=I_{p}+2m_{\omega }\left(2b^{2}+l^{2}\right)+4I_{\omega }+m_{p}\left({f_{1}}^{2}+{f_{2}}^{2}\right),m_{13}=m_{p}\left(-f_{2}\sin\theta +f_{1}\cos\theta \right)-2m_{\omega }l\sin\theta ,\\ m_{23}=m_{p}\left(f_{2}\cos\theta +f_{1}\sin\theta \right)+2m_{\omega }l\cos\theta ,\\ v_{13}=-2m_{\omega }l\;\dot{\theta }\;\cos\theta +m_{p}\dot{\theta }\;(-f_{2}\cos\theta -f_{1}\sin\theta ),\\ v_{23}=m_{p}\dot{\theta }(-f_{2}\sin\theta +f_{1}\cos\theta )-2m_{\omega }l\;\dot{\theta }\sin\theta . \end{array}$$

It would be more suitable to express the dynamic equations of motion in terms of internal velocities. By substituting (1) and its differentiation into Equation (8) and multiplying by $J^{T}(q)$, we can obtain

(10) $$\bar{M}(q)\dot{V}+\bar{C}(q,\dot{q})V=\bar{N}(q)\tau +{\bar{\delta }}_{d}$$

where $\bar{M}\in R^{2\times 2}=J^{T}M\;J,\bar{C}\in R^{2\times 2}=J^{T}[M\dot{J}+VJ],\bar{N}\in R^{2\times 2}=J^{T}N$, ${\bar{\delta }}_{d}\in R^{2}$ is the lumped system disturbance, which embraces external disturbances and model uncertainties. The elements in (10) are defined as follows:

$$\begin{array}{c} \bar{M}(q)=\left[\begin{array}{cc} m+I\theta \frac{\tan\varphi^{2}}{l}+\frac{8I\omega }{r^{2}}+2\frac{\tan\varphi }{l}(m_{13}\cos\theta +m_{23}\sin\theta ) & \frac{2I_{\omega }\tan\varphi }{l}\\ \frac{2I_{\omega }\tan\varphi }{l} & 2I_{\omega } \end{array}\right]\\ \bar{C}(q,\dot{q})=\left[\begin{array}{cc} c11 & c12\\ c21 & c22 \end{array}\right],\bar{N}(q)=\left[\begin{array}{cc} 1+l\sin^{2}\varphi +\frac{1}{r} & 0\\ 0 & 1 \end{array}\right]\\ c11=\frac{\tan\varphi }{l}(-\dot{\theta }m_{13}\sin\theta +\dot{\theta }m_{23}\cos\theta +v_{13}\cos\theta +v_{23}\sin\theta )+(m_{13}\cos\theta +m_{23}\sin\theta )\frac{\dot{\varphi }}{l\cos^{2}\varphi }+\frac{I_{\theta }\dot{\varphi }\tan\varphi }{l^{2}\cos^{2}\varphi },\\ c12=0,c21=\frac{2I_{\omega }\dot{\varphi }}{l\;\cos^{2}\varphi },c20=0 \end{array}$$

**Assumption** **1.** *The lumped disturbance* ${\bar{\delta }}_{d}$ *has a bounded first derivative; there exists a positive constant D such that* $|{\bar{\delta }}_{d}|\leq D$.

**Lemma** **1.** *For a nonlinear system* $\dot{x}=f(x(t))$*,* $x\in R^{n},x(0)=x_{0}$*, where* $f:R^{n}\to R^{n}$*, if there is a continuous positive definite function V (x) [26,40] satisfying:*

(11) $$\dot{V}(x)⩽-(\alpha V{\left(x\right)}^{p}+\beta V{\left(x\right)}^{q})$$

*where α > 0, β > 0, 0 < p < 1, q > 1, then the system can be stable in a fixed time, and the convergence time satisfies the following:*

(12) $$t<T\max=\frac{1}{\alpha (1-p)}+\frac{1}{\beta (q-1)}$$

## 3. Design of the Controllers

**Figure 2:**
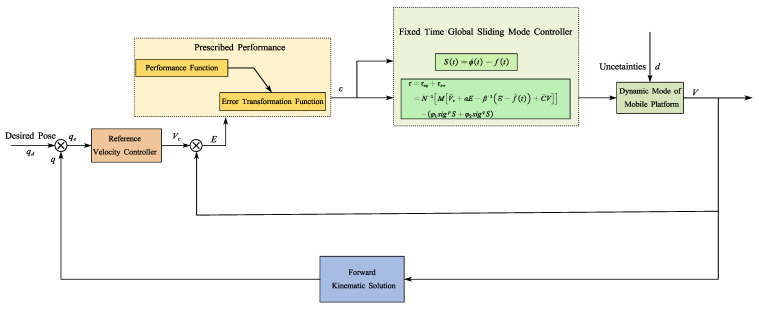
Prescribed performance fixed-time global sliding mode control system structure diagram.

This section will introduce the design of a fixed-time global sliding mode controller with prescribed performance for the trajectory tracking control of a mobile platform. First, a speed controller is designed based on the kinematic model of the mobile platform to provide input for the design of the dynamic controller; then, a constraint conversion function is used to convert the input error into an equivalent unconstrained error, and a global sliding mode surface is designed based on the unconstrained error, and the dynamic controller is designed in conjunction with the fixed-time theorem. A control block diagram of the mobile platform of SDPR is shown in Figure 2:

### 3.1. Kinematic Controller Design for the Mobile Platform of SDPR

Defining the reference trajectory pose of the mobile platform $\eta_{d}={\left[x_{d}\;y_{d}\;\theta_{d}\right]}^{T}$, and its actual trajectory pose $\eta ={\left[x\;y\;\theta \right]}^{T}$, the pose tracking error η_e_ between the expected trajectory and the actual trajectory can be obtained:

(13) $$\eta_{e}=\left[\begin{array}{c} x_{e}\\ y_{e}\\ \theta_{e} \end{array}\right]=\left[\begin{array}{ccc} \cos\theta & \sin\theta & 0\\ -\sin\theta & \cos\theta & 0\\ 0 & 0 & 1 \end{array}\right]\left[\begin{array}{c} x_{d}-x\\ y_{d}-y\\ \theta_{d}-\theta \end{array}\right]$$

The derivative of (13) is as shown:

(14) $${\dot{\eta }}_{e}=\left[\begin{array}{c} {\dot{x}}_{e}\\ {\dot{y}}_{e}\\ {\dot{\theta }}_{e} \end{array}\right]=\left[\begin{array}{c} v_{d}\cos\theta_{e}-v+y_{e}\omega \\ v_{d}\sin\theta_{e}-x_{e}\omega \\ \omega_{d}-\omega \end{array}\right]$$

The ${\dot{\eta }}_{d}$ is defined as

(15) $${\dot{\eta }}_{d}=\left[\begin{array}{c} {\dot{x}}_{d}\\ {\dot{y}}_{d}\\ {\dot{\theta }}_{d} \end{array}\right]=\left[\begin{array}{c} \cos\theta \cdot v_{d}\\ \sin\theta \cdot v_{d}\\ \omega_{d} \end{array}\right]$$

The stability and convergence of this controller are described through the choice of a Lyapunov function candidate *V*_0_.

(16) $$V_{0}=\frac{1}{2}\left(x_{e}^{2}+y_{e}^{2}\right)+\frac{1-\cos\theta_{e}}{k_{y}}\geq 0$$

Its derivative gives

(17) $${\dot{V}}_{0}={\dot{x}}_{e}x_{e}+{\dot{y}}_{e}y_{e}+{\dot{\theta }}_{e}\frac{\sin\theta_{e}}{k_{y}}$$

The substitution of Equation (15) into (17) provides the following:

(18) $$\begin{array}{l} {\dot{V}}_{0}=(v_{d}\cos\theta_{e}-v+y_{e}\omega )x_{e}+(v_{d}\sin\theta_{e}-x_{e}\omega )y_{e}+(\omega_{d}-\omega )\frac{\sin\theta_{e}}{k_{y}}\\ \;=x_{e}v_{d}\cos\theta_{e}-x_{e}v+y_{e}v_{d}\sin\theta_{e}+\frac{\sin\theta_{e}(\omega_{d}-\omega )}{k_{y}}\\ \;=x_{e}(v_{d}\cos\theta -v)+\sin\theta_{e}(\frac{\left(\omega_{d}-\omega \right)}{k_{y}}+y_{e}v_{d}\sin\theta_{e}) \end{array}$$

To satisfy the Lyapunov stability condition, the controller for the kinematic control in the outer loop is designed, which provides the reference input $V_{C}={\left[v_{c}\;,\omega_{c}\right]}^{T}$ for the dynamic controller:

(19) $$\left[\begin{array}{c} v_{c}\\ \omega_{c} \end{array}\right]=\left[\begin{array}{c} v_{d}\cos\theta_{e}+k_{x}x_{e}\\ k_{y}y_{e}v_{d}+\omega_{d}+k_{\theta }\sin\theta_{e} \end{array}\right]$$

And, we obtain ${\dot{V}}_{0}=-\frac{k_{\theta }}{k_{y}}\sin^{2}\theta_{e}-k_{x}x_{e}^{2}\leq 0$. Where *k_x_*, *k_y_*, and *k_θ_* are positive constants, and *v*_c_ and ω_c_ are reference linear and angular velocities, respectively.

### 3.2. Dynamic Controller Design for the Mobile Platform of SDPR

In this section, the control goal of the inner loop dynamics controller is to make the actual speed of the mobile platform $V={\left[v\;,\omega \right]}^{T}$ and track the expected speed $V_{C}={\left[v_{c}\;,\omega_{c}\right]}^{T}$ in a fixed time.

We construct a speed error function $E\;(t)={\left[e_{v}\;,e_{\omega }\right]}^{T}$ based on the actual speed and expected speed of the mobile platform:

(20) $$\begin{array}{l} e_{v}\;\;\;=v_{c}-v\\ e_{\omega }=\omega_{c}-\omega \end{array}$$

**Figure 3:**
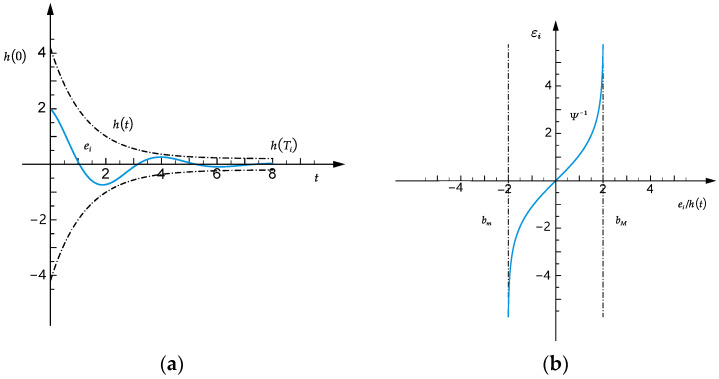
(**a**) Performance boundaries. (**b**) Error transformation function mapping diagram.

In this paper, the prescribed performance control is used to ensure that tracking errors converge to any prescribed small residual set [41], as shown in Figure 3a. At the same time, the maximum convergence speed and minimum overshoot are guaranteed. The prescribed boundaries for tracking errors are as follows:

(21) $$b_{m}\;h(t)<e_{i}<b_{M}\;h(t)$$

where $h\;(t)=\left\{\begin{array}{ll} \left(h\;(0)-h\;(T_{i})\right){\left(1-\frac{t}{T_{i}}\right)}^{l_{i}}+h\;(T_{i}) & t\in [0,T_{i})\\ h\;(T_{i}) & t\in [T_{i},+\infty ) \end{array}\right.$, $b_{m}=\left\{\begin{array}{cc} -\vartheta & e_{i}(0)⩾0\\ -1 & e_{i}(0)<0 \end{array}\right.$, $b_{M}=\left\{\begin{array}{cc} 1 & e_{i}(0)⩾0\\ \vartheta & e_{i}(0)<0 \end{array}\right.$, *i* as *v*, *ω*, 0 < *ϑ* < 1 is the overshoot index constant, *h* (t) is a prescribed performance function, and *l_i_* < 2 are defined positive constants.

Under the constrained tracking errors condition (21), designing the controller is inconvenient. To facilitate controller design, the constrained tracking errors must be converted into the equivalent unconstrained errors. Therefore, an error transformation function is designed as follows:

(22) $$\psi (x)=\frac{b_{m}b_{M}(\exp(x)-1)}{b_{m}\exp(x)-b_{M}}$$

The inverse function of (22) is the error conversion function that maps the constrained space to an unconstrained space $\psi^{-1}(x)=\ln\left(\frac{b_{M}(x-b_{m})}{-b_{m}(b_{M}-x)}\right)$, as shown in Figure 3b.

The unconstrained error after conversion is as follows:

(23) $$\varepsilon_{i}=\psi^{-1}\left(\frac{e_{i}}{h}\right)$$

The differential of Equation (23) is

(24) $${\dot{\varepsilon }}_{i}=\beta_{i}(\dot{e}+\alpha_{i}e_{i})$$

where $\alpha_{i}=-\frac{\dot{h}}{h}$, $\beta_{i}=\frac{1}{e-hb_{m}}+\frac{1}{hb_{M}-e}$, and *i* represents *ε* and *ω*.

For the mobile platform system above, a global sliding mode surface is designed as shown:

(25) $$S(t)=\phi (t)-f(t)=\left[\begin{array}{c} r_{v}\varepsilon_{v}+k_{v}\int_{0}^{t}\varepsilon_{v}(\tau )d\tau \\ r_{\omega }\varepsilon_{\omega }+k_{\omega }\int_{0}^{t}\varepsilon_{\omega }(\tau )d\tau \end{array}\right]-f(t)$$

To obtain the global sliding surface, *f* (t) needs to satisfy the following three conditions:

(1) f(0)=ϕ(0);
(2) $t\to \infty ,f\left(t\right)\to 0$;
(3) $f\left(t\right)$ has the first-order derivative.

Therefore, *f* (t) is usually designed as a monotonic exponential decay function as follows:

(26) $$f(t)=f(0)e^{-k\;t}$$

where k is the positive value.

From Equation (25), when *S* (0) = 0, the initial state of the system can remain on the sliding mode surface. Therefore, the reaching phase in the SMC is eliminated by this method, ensuring the global robustness of the system. Meanwhile, with the derivative $\dot{S}(t)$ of the sliding surface *S* (t), one can obtain that the following:

(27) $$\dot{S}(t)=\dot{\phi }(t)-\dot{f}(t)=0$$

Bringing Equations (15) and (22) into the above equation allows us to obtain the equivalent control law:

(28) $$\tau_{eq}=N^{-1}\left[M[{\dot{V}}_{c}+\beta^{-1}\alpha E\beta^{-1}R^{-1}(\Xi -\dot{f(t)})]+\bar{C}V\right]$$

where $\alpha =diag(\alpha_{v}\;,\alpha_{\omega }),\beta =diag(\beta_{v}\;,\beta_{\omega }),R=diag(r_{v}\;,r_{\omega }),\Xi ={\left[\varepsilon_{v}\;,\varepsilon_{\omega }\right]}^{T}$. The switching control term is designed to offset the effects of system’s model uncertainties and external disturbances. The control law is introduced as follows:

(29) $$\begin{array}{l} \tau =\tau_{eq}+\tau_{sw}\\ \;\;\;\;=N^{-1}\left[M\left[{\dot{V}}_{c}+\alpha E-\beta^{-1}(\Xi -\dot{f}(t))\right]+\bar{C}V\right]-(\varphi_{1}sig^{p}S+\varphi_{2}sig^{q}S) \end{array}$$

where *φ*_1_ and *φ*_2_ are positive constants, $sig^{r}x={\left|x\right|}^{r}sig(x)$, 0 < *p* < 1, *q* > 1.

**Theorem** **1.** *The parallel robot mobile platform control system with global robustness is stable at a fixed time, which can converge to the equilibrium point in a fixed time independent of the initial conditions of the system. The settling time is upper bounded by*

(30) $$T\leq \frac{1}{\varphi_{1}2^{\frac{p+1}{2}}(1-\frac{p+1}{2})}+\frac{1}{\varphi_{2}2^{\frac{q+1}{2}}(\frac{q+1}{2}-1)}$$

**Remark** **1.** *A model of the dynamics of a varying center of mass mobile platform using the Lagrange method and the controller (29) can be obtained by designing a global sliding mode surface (25). The controller improves the transient performance of the system by constraining the error characteristics through the performance function, which was not considered in reference [33].*

**Proof** **of Theorem 1.** Choose the Lyapunov candidate as:

(31) $$V=\frac{1}{2}S^{T}S$$

Combining the sliding mode function (25) and the control law (29) results in the time derivative of V, which can be obtained as follows:

(32) $$\begin{array}{cc} \dot{V} & =S^{T}\dot{S}\;\;\;\;\;\;\;\;\\ & =S(-\varphi_{1}sig^{p}S-\varphi_{2}sig^{q}S)\;\;\\ & =-\varphi_{1}{\left|\;S\;\right|}^{p+1}-\varphi_{2}{\left|\;S\;\right|}^{q+1}\;\;\;\;\;\;\;\\ & \leq -\varphi_{1}2^{\frac{p+1}{2}}V^{\frac{p+1}{2}}-\varphi_{2}2^{\frac{q+1}{2}}V^{\frac{q+1}{2}} \end{array}$$

Therefore, according to Lemma 1, the system will converge to the equilibrium point in a fixed time *t*_1,_

(33) $$t_{1}\leq \frac{1}{\varphi_{1}2^{\frac{p+1}{2}}(1-\frac{p+1}{2})}+\frac{1}{\varphi_{2}2^{\frac{q+1}{2}}(\frac{q+1}{2}-1)}$$

□

## 4. Discussion and Analysis of Simulation Results

To validate the proposed method, this section presents and analyzes two sets of comparative simulation experiments.

1. Finite-time global sliding mode control (FnTGSMC) is compared with fixed-time global sliding mode control (FxTGSMC).
2. Fixed-time global sliding mode control (FxTGSMC) is compared with the prescribed performance fixed-time global sliding mode control (PPFxTGSMC).

The model parameters of the parallel robot mobile platform for sandblasting and rust removal are shown in Table 1.

In the simulation comparison experiment, the parameters of the mobile platform model of SDPR are consistent; the initial position of the mobile platform $q_{0}={\left[0.2\;,0.2,\pi /4\right]}^{T}$ and the lumped interference ${\bar{\delta }}_{d}=5\cos(2t)+2\sin(1.5t)$ are also consistent.

Define the origin of the global coordinate system as a point close to the steel box girder, the X-axis as the direction parallel to the steel box girder, and the Y-axis as the direction of the vertical steel box girder. Two sets of experiments were designed to ensure accurate results. In Case I, the desired trajectory of the mobile platform is given:

(34) $$x_{d}=\left\{\begin{array}{ll} 0.06\;t^{2} & \left(0\text{–}1s\right)\\ 0.06+0.12(t-1) & \left(1\text{–}14s\right)\\ 1.62+0.12(t-14)-0.06{\left(t-14\right)}^{2} & \left(14\text{–}15s\right)\\ 1.68 & \left(15\text{–}16s\right)\\ 1.68-0.06{\left(t-16\right)}^{2} & \left(16\text{–}17s\right)\\ 1.62-0.12(t-17) & \left(17\text{–}30s\right)\\ 0.06-0.12(t-30)+0.06{\left(t-30\right)}^{2} & \left(30\text{–}31s\right) \end{array}\right.$$

In actual projects, the lifting mechanism will cause changes in the center of mass of the mobile platform during the movement of the mobile platform, and the change trajectory of the center of mass is related to the size of the mobile platform. In Case I, the centroid trajectory of the mobile platform is as follows:

(35) $$\left\{\begin{array}{ll} f_{1}=\frac{b}{2}\sin\left(\frac{\pi t}{2}\right)+\frac{l}{2} & \left(0⩽t<1\right)\\ f_{1}=\frac{b}{2}+\frac{l}{2} & \left(1⩽t<16\right)\\ f_{1}=\frac{b}{2}\cos\left(\frac{\pi (t-16)}{2}\right)+\frac{l}{2} & \left(16⩽t<17\right)\\ f_{1}=\frac{l}{2} & \left(17⩽t<31\right) \end{array}\right.$$

**Figure 4:**
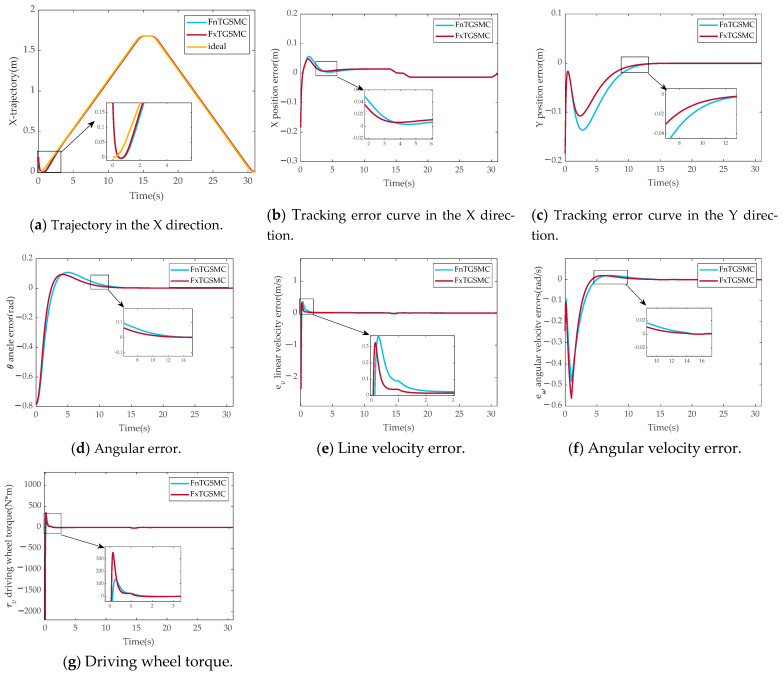
Results of simulation comparison experiments between FnTGSMC and FxTGSMC. (**a**) Trajectory in the X direction. (**b**) Tracking error curve in the X direction. (**c**) Tracking error curve in the Y direction. (**d**) Angular error. (**e**) Line velocity error. (**f**) Angular velocity error. (**g**) Driving wheel torque.

Figure 4 shows the results of the simulation comparison between finite-time global sliding mode control and fixed-time global sliding mode control.

Figure 4a–d show the results of the comparison of pose tracking errors between parallel robot mobile platforms with FnTGSMC and FxTGSMC. The settling times of the pose tracking errors with FnTGSMC are 4.39 s, 12.51 s, and 12.32 s, and with FxTGSMC, the settling times are reduced to 3.5 s, 11.93 s, and 10.91 s. It can be seen that the stability errors of FnTGSMC and FxTGSMC are almost the same, but the convergence speed of FxTGSMC is significantly faster than that of FnTGSMC.

Figure 4e shows that the linear velocity errors of FnTGSMC and FxTGSMC, respectively, converge in 2.70 s and 1.97 s. Figure 4f shows that the angular velocity errors of FnTGSMC and FxTGSMC, respectively, converge to near 0 in 13.20 s and 11.66 s. The comparison results show that the convergence speed of FxTGSMC is faster, but both FnTGSMC and FxTGSMC have large overshoots during convergence. Figure 4g shows that the maximum torque of the driving is lower for FnTGSMC than for FxTGSMC, but the convergence speed of FxTGSMC is faster. Therefore, it can be concluded that FxTGSMC has a faster convergence speed.

**Figure 5:**
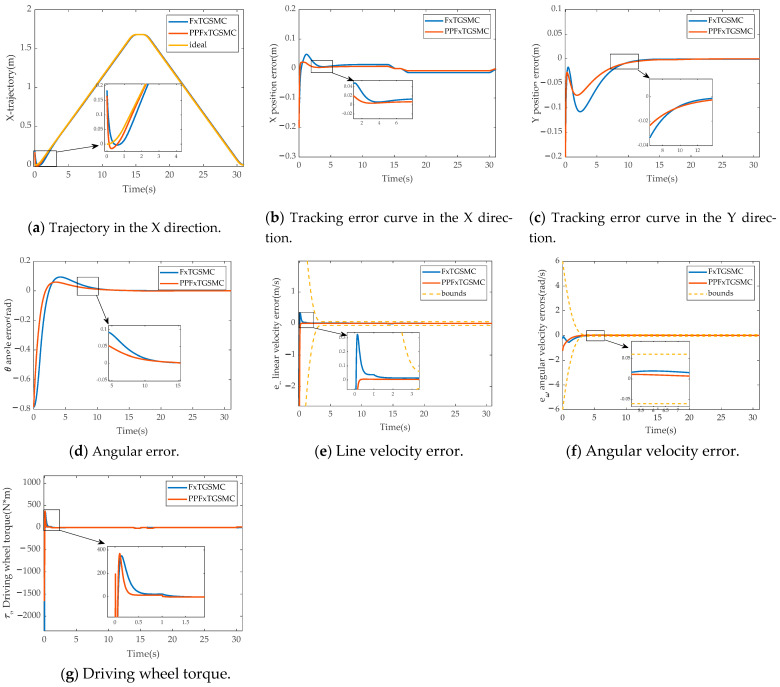
Comparison results of simulation experiments between FxTGSMC and PPFxTGSMC. (**a**) Trajectory in the X direction. (**b**) Tracking error curve in the X direction. (**c**) Tracking error curve in the Y direction. (**d**) Angular error. (**e**) Line velocity error. (**f**) Angular velocity error. (**g**) Driving wheel torque.

Figure 5 shows the simulation results of fixed-time global sliding mode control and fixed-time global sliding mode control with prescribed performance.

Figure 5a–d show the results of a comparison of the pose tracking errors of parallel robot mobile platforms between FxTGSMC and PPFxTGSMC. The results show that the maximum overshoot of the tracking error in the X direction reduces to 0.021 m from 0.048 m, and the maximum overshoot of the angular error reduces to 0.057 rad from 0.093 rad. Moreover, the convergence speed of PPFxTGSMC is faster.

As shown in Figure 5e, the linear velocity error of FxTGSMC reaches a maximum overshoot of 0.32 m/s in 0.14 s and converges to near zero in 1.72 s; the linear velocity error of PPFxTGSMC converges to near zero in 0.35 s without overshoot. In Figure 5f, the angular velocity error of FxTGSMC reaches a maximum overshoot of 0.019 m/s in 5.97 s and converges to near zero in 10.13 s; then, PPFxTGSMC converges to near zero in 4.82 s without the overshoot. Figure 5g shows that FxTGSMC reduces the setting time of driving wheel torque from 1.52 N*m to 1.09 N*m. Therefore, it can be concluded that PPFxTGSMC can eliminate the overshoot of angular velocity tracking errors and linear velocity tracking errors on mobile platforms.

In Case II, the parameters of the three controllers remain unchanged, the total error of the mobile platform changes as ${\bar{\delta }}_{d}=4\sin(t)+1.5\cos(2.5t)$, and the desired trajectory of the mobile platform is as follows:

(36) $$x_{d}=\left\{\begin{array}{ll} 0.05t^{2} & \left(0\text{–}1s\right)\\ 0.05+0.12(t-1) & \left(1\text{–}15s\right)\\ 1.73+0.12(t-15)-0.06{\left(t-15\right)}^{2} & \left(15\text{–}16s\right)\\ 1.79 & \left(16\text{–}31s\right) \end{array}\right.$$

The centroid trajectory of the mobile platform is as follows:

(37) $$\left\{\begin{array}{ll} f_{1}=\frac{b}{3}\sin\left(\frac{\pi t}{2}\right)+\frac{l}{4} & \left(0⩽t<1\right)\\ f_{1}=\frac{b}{3}+\frac{l}{4} & \left(1⩽t<16\right)\\ f_{1}=\frac{b}{3}\cos\left(\frac{\pi (t-16)}{2}\right)+\frac{l}{4} & \left(16⩽t<17\right)\\ f_{1}=\frac{l}{4} & \left(17⩽t<31\right) \end{array}\right.$$

(38) $$\left\{\begin{array}{ll} f_{2}=-\frac{2b}{5}\cos\left(\frac{\pi t}{2}\right)+\frac{2b}{5} & \left(0⩽t<1\right)\\ f_{2}=\frac{2b}{5} & \left(1⩽t<16\right)\\ f_{2}=\frac{2b}{5}\sin\left(\frac{\pi (t-16)}{2}\right)+\frac{2b}{5} & \left(16⩽t<17\right)\\ f_{2}=\frac{4b}{5} & \left(17⩽t<31\right) \end{array}\right.$$

**Figure 6:**
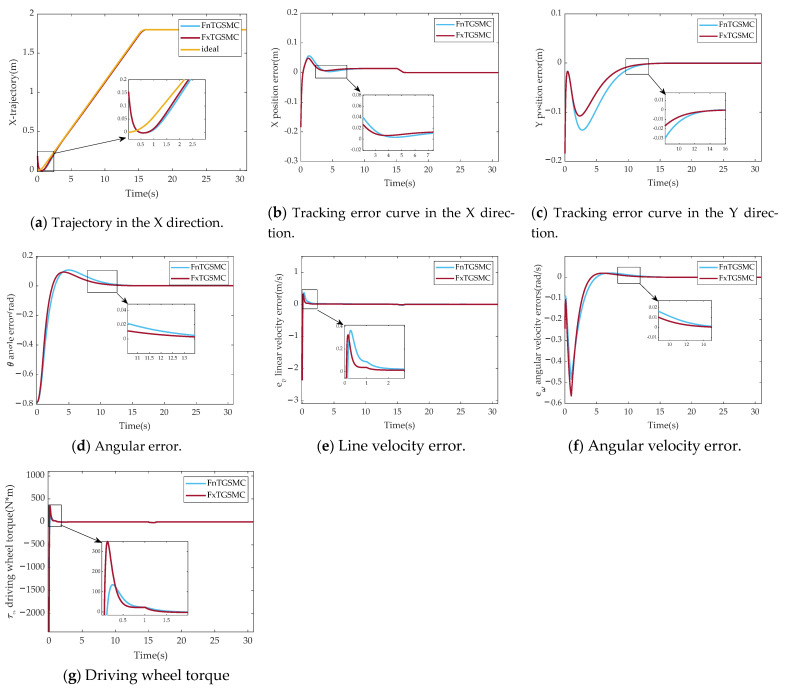
Results of comparison of simulation experiments between FnTGSMC and FxTGSMC. (**a**) Trajectory in the X direction. (**b**) Tracking error curve in the X direction. (**c**) Tracking error curve in the Y direction. (**d**) Angular error. (**e**) Line velocity error. (**f**) Angular velocity error. (**g**) Driving wheel torque.

Figure 6 shows the simulation comparison results between finite-time global sliding mode control and fixed-time global sliding mode control in Case II.

Figure 6a–d show a comparison of the results for the pose tracking errors of parallel robot mobile platforms with FnTGSMC and FxTGSMC. The settling times of the pose tracking errors of FnTGSMC are 8.58 s, 13.31 s, and 13.26 s, and with FxTGSMC, the settling times are reduced to 7.85 s, 13.14 s, and 12.1 s.

The results shown in Figure 6e are similar to those in Case I, the linear velocity errors of FnTGSMC and FxTGSMC, respectively, converge in 2.88 s and 1.8 s. Figure 4f shows that the angular velocity errors of FnTGSMC and FxTGSMC, respectively, converge to near 0 in 13.39 s and 11.71 s. Figure 6g shows that FxTGSMC has a faster convergence speed. Therefore, it can be concluded that fixed-time control has a faster convergence speed than finite-time control.

**Figure 7:**
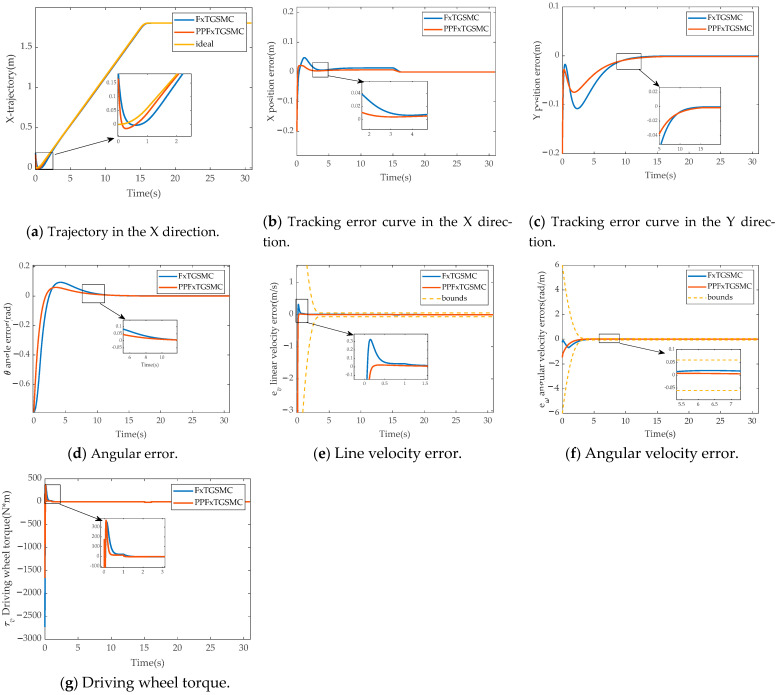
Results of comparison of simulation experiments between FxTGSMC and PPFxTGSMC. (**a**) Trajectory in the X direction. (**b**) Tracking error curve in the X direction. (**c**) Tracking error curve in the Y direction. (**d**) Angular error. (**e**) Line velocity error. (**f**) Angular velocity error. (**g**) Driving wheel torque.

Figure 7 shows the simulation results of fixed-time global sliding mode control and fixed-time global sliding mode control with the prescribed performance in Case II.

In Figure 7a, the maximum overshoot in the FxTGSMC and PPFXTGSMC schemes is 0.047 m and 0.022 m; in Figure 7d, the maximum overshoot of both schemes is 0.09 m and 0.053 m; in Figure 7e, the maximum overshoot of FxTGSMC is 0.324 m/s and that of PPFXTGSMC is 0.021m/s; in Figure 7f, the maximum overshoot of FxTGSMC is 0.018 rad/s, and PPFXTGSMC has no overshoot. Therefore, it can be concluded that prescribed performance control can eliminate the overshoot of system errors.

Compared with finite-time control, fixed-time control can provide faster convergence performance and the convergence time of fixed-time control does not depend on the initial state of the system; the prescribed performance control can constrain the convergence characteristics of the system error and reduce the chance pf overshooting the system error; the control method in this paper is based on the global sliding mode design, which improves the global robustness of the system. The above simulation results show that the PPFxTGSMC algorithm can reduce the chance of overshooting the trajectory tracking error in a parallel robotic mobile platform with model uncertainty and external disturbance and ensure the system converges at a fixed time, which effectively improves the performance of the trajectory tracking control of the parallel robotic mobile platform.

## 5. Conclusions

In this work, we propose a fixed-time global sliding mode control method with a prescribed performance to address the issue of trajectory tracking control in a parallel robot mobile platform with model uncertainties and external disturbances. This method eliminates the reaching phase of the sliding mode using an auxiliary function and improves the convergence performance of the system in combination with fixed-time control. To improve the transient performance of the system, we design a performance function that constrains the tracking error characteristics of the mobile platform within a prescribed boundary. Utilizing Lyapunov stability theory, we rigorously prove the fixed-time stability of the proposed controller. Simulation experiments substantiate the effectiveness of the control method. In the future, an intelligent method for the optimization of the control parameters will be investigated.

## Figures and Tables

**Table 1:** Model parameters of mobile platform for SDPR.

Parameters	Value
*m_p_*/kg	780
*m_ω_*/kg	5
*I_p_*/kg·m^2^	9.45
*I_ω_*/kg·m^2^	0.017
*r*/m	0.125
*l*/m	1.23
*b*/m	0.37

## Data Availability

Data are contained within the article.
